# Analysis of sugars and amino acids in aphid honeydew by hydrophilic interaction liquid chromatography – Mass spectrometry

**DOI:** 10.1016/j.mex.2020.101050

**Published:** 2020-09-02

**Authors:** Phuong K. Nguyen, Janel E. Owens, Luis E. Lowe, Emily H. Mooney

**Affiliations:** a Department of Chemistry and Biochemistry, University of Colorado Colorado Springs, United States; b Department of Biology, University of Colorado Colorado Springs, United States

**Keywords:** Honeydew composition, HILIC, LC/MS, Melezitose, Raffinose, *Aphis asclepiadis*

## Abstract

Past analyses of sugar and amino acid composition of aphid honeydews have been completed using diverse instrumentation. Here we report the use of hydrophilic interaction liquid chromatography (HILIC) coupled with a triple quadrupole mass spectrometric (MS/MS) detector for the analysis of seven saccharides (xylose, fructose, glucose, sucrose, trehalose, melezitose and raffinose) and five amino acids (glutamic acid, glutamine, aspartic acid, serine, and asparagine). Limits of quantitation ranged from 0.05 mg/L (melezitose) to 1.0 mg/L (fructose) for sugars and from 0.10 mg/L (glutamic acid) to 3.66 mg/L (asparagine) for amino acids. Sample preparation was fast and simple, requiring only the washing of foils used to collect aphid honeydew with hot (80 °C) water and sonication of samples prior to HILIC/MS/MS analysis for both classes of analytes. No analyte derivatization was required and excellent chromatographic characteristics were observed. For those studying honeydew-mediated interactions in the field, this technique allows for rapid characterization of ecologically important amino acids and sugars.

• Composition of seven saccharides in *Aphis asclepiadis* honeydew including xylose, fructose, glucose, sucrose, trehalose, melezitose,and raffinose, and five standard amino acids including glutamic acid, glutamine, aspartic acid, serine, and asparagine, were analyzed using hydrophilic interaction liquid chromatography-mass spectrometry.
• All polar analytes were analyzed without derivatization using HILIC-MS with chromatographic run times of 7 min (sugars) and 10 min (amino acids).

## Graphical abstract

**Figure:**
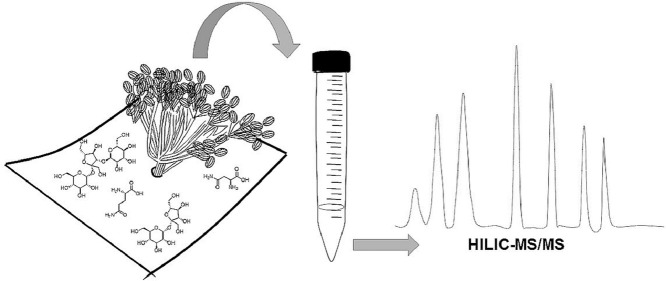

Specifications Table

**Table:** 

Subject Area:	Chemistry
More specific subject area:	Analytical chemistry, sugar, amino acids
Method name:	Sugar and Amino Acid Determination by HILIC-MS
Name and reference of original method:	M.K. Fisher, A. W. Shingleton, “Host plant and ants influence the honeydew sugar composition of aphids.” **2001**, *15* (4), 544–550. https://doi.org/10.1046/j.0269-8463.2001.00550.x
Resource availability:	*n/a*

## Introduction

Ant-aphid mutualisms are keystone interactions in a variety of communities [1,2]. In these interactions, ants provide protection from predators in exchange for aphid honeydew, a sugar-rich food source that also contains trace amounts of amino acids. Honeydew composition is an important driver of ant-aphid interactions as well as interactions with predators and parasitoids [3,4]. A recent study reported how aphid honeydew composition affects longevity and fecundity on hyperparasitoid wasps, a fourth trophic level insect [5]. Total sugar content in honeydew can be characterized using colorimetric anthrone-sulfuric acid assays [6] though other analytical techniques provide more detailed information on the composition of specific sugars. Aphid honeydew composition is typically analyzed via high performance liquid chromatography (HPLC) coupled with specific detectors capable of detecting sugars or amino acids, such as an evaporative light scattering detector (ELSD), pulsed amperometric detection (PAD), refractive index detector (RID), or electrochemical detector (ECD) (Table 1).

**Table 1:** Survey of recent literature for instrumentation used for the analysis of aphid honeydew.

Analytes	Detector	Chromatographic run time (min)	Limit of detection (LOD)	Reference
Sugars: arabinose, xylose, rhamnose, fructose, glucose, sucrose, maltose, trehalose, trehalusose, melezitose, raffinose, stachyose, turanose, erlose	HPLC with ECD	30	Not reported	Fischer and Shingleton [4]
Sugars: fructose, glucose, arabinose, sucrose	HPLC with RID	Not reported	Not reported	Golan and Najda [7]
Sugars and amino acids	UPLC with ELSD (sugars); HPLC with photodiode array (derivatized amino acids)	9 (sugars); 53 (amino acids)	Not reported	Pringle et al. [8]
Sugars: sorbitol, mannitol, trehalose, glucose, fructose, melibiose, sucrose, melezitose, raffinose, maltose	HPLC with ECD	35	2.5 ppm	van Neerbos et al. [5]
Sugars: glucose, fructose, sucrose, trehalose, melibiose, maltose, isomaltose, maltulose, isomaltulose, melezitose, erlose, raffinose, 1-kestose, isomaltotriose, maltotriose, nidrose, stachyose	HPLC with anion exchange column and pulse amperometric detection	25	Not reported	Shaaban et al. [9,10]

Hydrophilic interaction liquid chromatography (HILIC) is an analytical technique for the separation of polar molecules in diverse samples. It offers improved chromatographic characteristics, such as better baseline separation and peak shape, when compared to reverse phase ion-pairing chromatography for polar molecules in complex sample matrices [11]. Here, we describe a method utilizing HILIC coupled with a triple quadrupole mass spectrometer for separation and detection of polar molecules (sugars and amino acids) from aphid honeydew. Use of HILIC-MS results in short chromatographic run time (7 min for sugars; 10 min for amino acids) excluding column re-equilibration with excellent limits of detection.

## Materials and methods

### Chemicals and reagents

Stock solutions of sugars (xylose, fructose, glucose, sucrose, trehalose, melezitose, and raffinose) and amino acids (L-glutamic acid, L-glutamine, L-aspartic acid, L-serine, and L-asparagine; all at ≥ 98% purity) were prepared from standards purchased from the following vendors: Fisher Scientific (Fair Lawn, NJ), Sigma-Aldrich (St. Louis, MO), and Macron Chemicals (Phillipsburg, NJ). LC-grade 18 MΩ organic-free water from a Barnstead filtration system (Fisher Scientific) was used for preparation of all standards, field collected samples, and the LC mobile phases. Optima LC/MS-grade solvents including methanol and acetonitrile were purchased from Fisher Scientific. MS-grade mobile phase modifiers ammonium hydroxide (≥ 25% assay) and ammonium formate (≥ 99.0% purity) were from Fisher Scientific. Formic acid (≥ 98% purity) was from Honeywell Fluka (Muskegon, MI). All other chemicals were from Fisher Scientific unless otherwise specified.

### Preparation of standards

Individual stock solutions (~1000 mg/L) of each sugar and amino acid were prepared by dissolving ~25 mg of each analyte (Mettler XS64 balance) into 25 mL methanol/water (10/90, v/v). Two 25 mL working stock solutions (one at 50 mg/L containing all sugars; the second at 50 mg/L containing all amino acids) were prepared in methanol/water (10/90, v/v) from the individual stock solutions. These working stock solutions were used to prepare two sets of calibration standards (one for sugars, the other for amino acids). In each calibration standard set, there were five 1-mL calibration standards: 10, 5, 2.5, 1, and 0.5 mg/L. The 5 mg/L calibration standard was serially diluted to create additional standards of 0.25, 0.10, and 0.05 mg/L, which were also at 1 mL volume. All calibration standards were prepared in methanol/water (10/90, v/v) and stored at 4 °C until analysis. Individual sugar and amino acid standards for MS optimization (~ 1 mg/L) were prepared by dissolving 1 μL of the ~1000 mg/L stock solution into 1 mL methanol/water (10/90, v/v).

### LC/MS system

A Shimadzu LCMS-8030 system was used for chromatographic separation and detection of sugars and amino acids. This system consisted of a binary solvent delivery system equipped an in-line mobile phase degasser, 15 °C autosampler, thermostat-controlled column oven, and triple quadrupole mass spectrometer fitted with an electrospray ionization (ESI) source. The ESI source was operated with the following conditions: nebulizing gas flow, 1.5 L/min; drying gas flow, 15 L/min; DL temperature 250 °C, and heat block temperature, 400 °C. HILIC separation for sugars and amino acids was performed on an Xbridge Amide column (3.0 mm x 100 mm, 3.5 μm, Waters Corporation, Milford, MA).

### MS optimization conditions

*Sugars:* Working solutions (~1 mg/L) of each sugar in methanol/water (10/90, v/v) were used for optimization of LC and MS conditions. Oligosaccharides contain a large number of hydroxyl groups, which can be detected by both positive and negative ionization modes when using ESI. To determine the preferred ionization mode, 1 μL of each one mg/L sugar solution was injected with the column removed from the LC/MS system while the ESI source was operated in dual polarity mode. Higher signal intensity was observed with negative ionization mode. Determination of the optimal ion (*m/z;* Table 2) for selected ion monitoring (SIM) mode for each sugar was performed by injecting 1 μL of 1 mg/L solution for each sugar using the Shimadzu software *LabSolutions* (v5.6 SP2) Optimization Wizard function.

**Table 2:** Selected ions (*m/z*) for seven sugars analyzed from aphid honeydew.

Sugar name, MW (g/mol)	Selected [M-H]^−^ ion (*m/z*)	Dwell time (ms)
Xylose, 150.13	149.0	60
Fructose, 180.16	179.0	60
Glucose, 180.16	179.0	60
Sucrose, 342.3	341.0	60
Trehalose, 342.3	341.0	60
Melezitose, 504.4	503.0	60
Raffinose, 504.4	503.0	60

*Amino acids:* Working solutions (~ 1 mg/L) of each amino acid in methanol/water (10/90, v/v) were prepared to determine optimal MS conditions. For determination of preferred ionization mode, 1 μL of each 1 mg/L amino acid solution was injected into the LC/MS system with column removed while the ESI source was operated in dual polarity mode. Here, positive ion mode resulted in improved signal intensity. Determination of optimal precursor ions (*m/z*), collision energy (V), Q1 and Q3 pre-bias rod voltages (V) and product ions (*m/z*) were optimized using the *LabSolutions* (v5.6 SP2) Optimization Wizard function. For each optimization parameter selected, 1 μL of each 1 mg/L amino acid solution was injected. Multiple reaction monitoring (MRM) was selected for glutamic acid, asparagine, and glutamine; and SIM was selected for serine and aspartic acid owing to poor product ion formation (Table 3).

**Table 3:** Precursor/product ion pairs for amino acids analyzed via MRM mode and SIM ions for serine and aspartic acid.

Amino acid	MS mode	Precursor or SIM ion, [M + H]^+^, *m/z*	MRM transition (precursor → product), *m/z*	Dwell time, ms	Q1 Pre-bias voltage, V	Collision Energy, V	Q3 Pre-bias voltage, V
Glutamic acid	MRM	148.05	148.05 → 83.95	13.0	−15.0	−21.0	−27.0
			148.05 → 101.75	13.0	−12.0	−13.0	−12.0
			148.05 → 56.20	13.0	−12.0	−31.0	−21.0
Asparagine	MRM	133.05	133.05 → 74.15	47.0	−21.0	−13.0	−16.0
Glutamine	MRM	147.05	147.05 → 83.95	22.0	−10.0	−19.0	−18.0
			147.05 → 56.00	22.0	−11.0	−30.0	−12.0
Serine	Q3 SIM	106.10	n/a	47.0	n/a	n/a	n/a
Aspartic acid	Q3 SIM	133.90	n/a	47.0	n/a	n/a	n/a

### Preparation of LC/MS mobile phases and gradient profiles

**Fig. 1:**
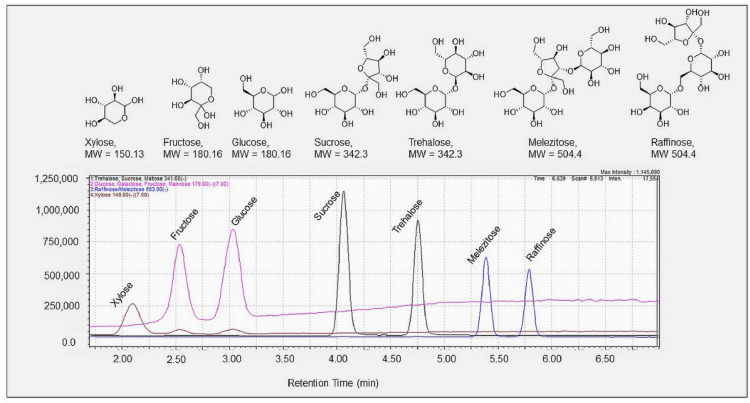
Structures and chromatogram of a 10.0 mg/L sugar standard with structures of seven sugars of interest: xylose, fructose, glucose, sucrose, trehalose, melezitose, and raffinose. All peaks exhibited baseline resolution with a resolving power of 1.83 or higher. The signal intensities for xylose, fructose, and glucose were magnified seven-fold here to illustrate the quality peak shape and resolution in a single figure.

*Sugars:* A Waters Corp. *Application Note* was used as a guide [12]. Mobile phase A consisted of acetonitrile/water (30/70, v/v) with 0.10% (v/v) ammonium hydroxide. Mobile phase B was acetonitrile/water (80/20, v/v) with 0.10% (v/v) ammonium hydroxide. A gradient elution was used to elute the seven sugars from the XBridge Amide column in under seven minutes: 0 min (100% B) ramped to 50% at 8 min. The column was returned to initial mobile phase conditions (100% B) at 8.2 min and re-equilibrated for 10.8 min prior to injection of the next standard or field sample. The flow rate was set at 0.6000 mL/min throughout the 20 min run. The column temperature was maintained at 35 °C. The injection volume for all standards and samples was 4 μL. Given that several sugars have the same molecular weight and hence *m/z* ratio (Table 2), eluting peaks needed to be fully resolved (*R* ≥ 1.5) (Fig. 1).

**Fig. 2:**
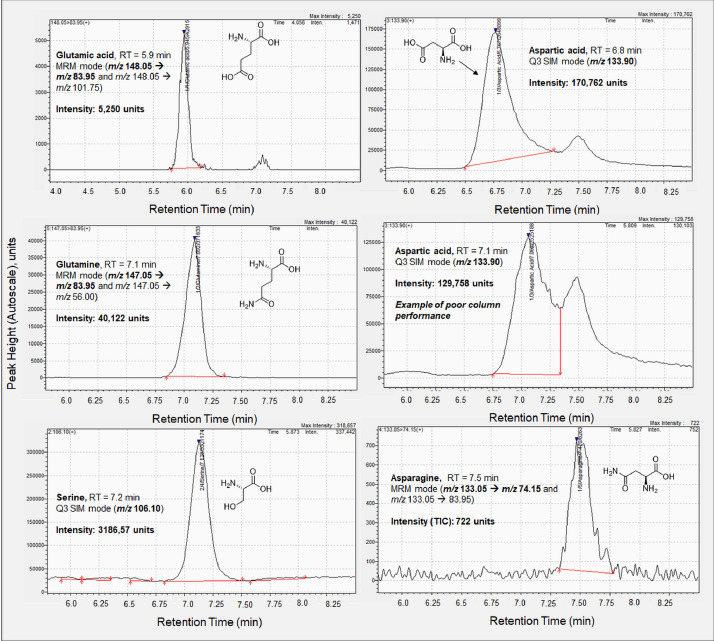
Integrated chromatogram of a 10.0 mg/L amino acid standard (data collected 02/15/2019) shown with structures of five amino acids of interest with quantitation transition or monitored ions shown in bold: glutamic acid (MRM mode), serine (Q3 SIM), aspartic acid (Q3 SIM), asparagine (MRM mode), and glutamine (MRM mode). Aspartic acid (RT = 6.8 min) exhibits a double peak in SIM, with the second peak coming from asparagine (RT = 7.5 min), which was analyzed via MRM. A second chromatographic trace of aspartic acid (middle panel, right column, data collected 1/8/2019) is provided to show an example of poor column performance. While the peak can still be integrated, as shown, aspartic acid serves as indicator of needed column care.

*Amino acids:* A previous method for the analysis of amino acids in fruit by HILIC-MS/MS [13] was used as a guide for optimizing LC and MS conditions on our Shimadzu LCMS-8030 system. Mobile phase A consisted of water with 0.15% (v/v) formic acid and 10 mM ammonium formate. Mobile phase B was acetonitrile with 0.15% formic acid. The gradient profile was as follows: 0 min (85% B) ramped to 80% B at 6 min, 70% B at 10 min, with a return to initial conditions (100% B) at 10.3 min with an 8.7 min re-equilibration time. The flow rate was 0.6000 mL/min throughout the 20 min run. The column was held at 35 °C and the injection volume was 4 μL. Amino acids were analyzed via MRM and SIM modes (Table 3). Note that there were two peaks in the chromatographic trace (Fig. 2) for aspartic acid (RT = 6.8 min), which was analyzed by SIM (*m/z* 133.90). The presence of a second peak in the aspartic acid chromatographic trace was expected because the Q1 and Q3 quadrupoles were operated with unit resolution. This second peak arises from analysis of asparagine (RT = 7.5 min), which was analyzed via MRM with a precursor ion *m/z* 133.05. Thus, the SIM ion for aspartic acid and the precursor ion for asparagine differed by 0.85 amu. Analysis of aspartic acid by MRM is preferred and possible owing to a similar fragmentation pattern to asparagine [14], but in our system, we were not able to achieve consistent product ion formation for aspartic acid. Two chromatographic traces for aspartic acid are shown in Fig. 2 to provide an example of poor column performance and the need for column care.

### Conditioning and care of HILIC column

Before the HILIC column was installed, LC pumps were purged at a flow rate of 1.000 mL/min with 100% LC-grade 18 MΩ water for 10 min followed by 100% LC/MS-grade acetonitrile for 10 min. The LC system was then flushed at 1.000 mL/min with a 50/50 (v/v) mixture of water/acetonitrile for 10 min prior to column installation. The column was flushed with 60/40 (v/v) acetonitrile/water for 1 hr at 0.6000 mL/min per the manufacturer's recommended conditioning procedure. The column was equilibrated for 25 min at 0.6000 mL/min with initial mobile phase conditions (100% mobile phase B for sugars analysis; 85% mobile phase B for amino acids analysis) prior to batch analyses. The peak shape of aspartic acid (Fig. 2) and its resolution from asparagine indicate the need for column care. To improve peak shape and resolving power of the column, the solvents were re-prepared and the column was flushed for 1 hr at 0.6000 mL/min with the initial column conditions for amino acid analyses.

### Preparation of calibration curves

External calibration curves were used for quantitation with a quadratic least squares fit with 1/*x* weighting. For sugars, calibration standards ranged from 0.05 to 10 mg/L with an R^2^ value of 0.9993 or higher (Table 4), while for amino acids, concentrations ranged from 0.10 to 10 mg/L with R^2^ values of 0.9989 or higher (Table 5). Calibration standards were analyzed at the beginning and end of every batch as well as throughout the batch with a calibration check occurring after every six field collected samples had been analyzed. The responses from all standards, including the ‘through-run’ standards, were included in the preparation of the calibration curves. Analytical figures of merit including *R^2^* values, linear range, limit of detection (LOD), and limit of quantitation (LOQ) are included in Tables 4 and 5.

**Table 4:** Analytical figures of merit for seven sugars analyzed from aphid honeydew by SIM.

Analyte, *m/z*	Retention Time (min)	Calibration curve	*R^2^*	Linear range (mg/L)	LOD (mg/L)	LOQ (mg/L)
Xylose, 149.0	2.51	*y* *=* 1285.33×^2^ + 135028x −6717.02	0.9993	0.75–10.0	0.25	0.75
Fructose, 179.0	3.10	*y* *=* 2507.07×^2^ + 168000x −1697.57	0.9995	1.0–10.0	0.33	1.0
Glucose, 179.0	3.55	*y* *=* *−*21.5830×^2^ + 208375x + 4149.39	0.9997	0.32–10	0.10	0.32
Sucrose, 341.0	4.25	*y* *=* *−*35622.9×^2^ + 1209780x −59960.2	0.9998	0.10–5.0	0.03	0.10
Trehalose, 341.0	5.26	*y* *=* *−*23908.5×^2^ + 948254x −10377.2	0.9999	0.14–5.0	0.05	0.14
Melezitose, 503.0	5.88	*y* *=* *−*12793.3×^2^ + 666895x +1938.22	0.9999	0.05–5.0	0.02	0.05
Raffinose, 503.0	6.22	*y* *=* *−*6962.58×^2^ + 521725x −3512.81	0.9997	0.22–5.0	0.07	0.22

**Table 5:** Analytical figures of merit for five amino acids analyzed from aphid honeydew.

Analyte, MS mode	Retention Time (min)	Calibration Curve	*R^2^*	Linear range (mg/L)	LOD (mg/L)	LOQ (mg/L)
Glutamic acid, MRM	5.9	*y* *=* 232.624×^2^ + 1931.87x *−*67.0475	0.9968	0.50–10.0	0.02	0.10
Asparagine, MRM	7.5	*y* *=* 29.5635×^2^ + 478.909x + 479.488	0.9983	3.66–10.0	1.21	3.66
Glutamine, MRM	7.1	*y* *=* 1436.62×^2^ + 22967.3x −170.907	0.9991	0.29–10	0.10	0.29
Serine, Q3 SIM	7.2	*y* *=* *−*5980.92×^2^ + 393826x −16030.8	0.9989	1.12–10	0.37	1.12
Aspartic acid, Q3 SIM	6.8	*y* *=* *−*3345.67×^2^ + 299378x +3371.20	0.9995	0.55–10	0.18	0.55

### Preparation of field collected samples of aphid honeydew for LC/MS analysis

Collection of aphid honeydew in the field was described previously [15]. Briefly, foil squares surrounding aphid-colonized inflorescences were left to collect honeydew for 24 hrs. After honeydew droplet-density was measured, a subsample of each foil was placed into 50 mL polypropylene centrifuge tubes. Two mL of 18 MΩ DI water heated to 80 °C were added to the centrifuge tube using an air displacement autopipette (Thermo Scientific). These centrifuge tubes were sonicated (FS20D sonicator, Fisher Scientific) for 15 min at 60 °C. One mL of each sample was transferred to an autosampler vial (Waters Corp) for direct analysis by LC/MS (sugars; selected amino acids) or LC/MS/MS (selected amino acids).

For samples with high concentrations of sugars or amino acids that were beyond the range of the calibration curve, samples were diluted 1:20 or 1:400. To make 1:20 diluted samples, 50 μL of the original honeydew extract was added to a new autosampler vial along with 950 μL of methanol/water (10/90, v/v) via a Hamilton gastight syringe. These samples were vortex mixed for 10 s prior to LC/MS analysis. The same dilution method was used to make 1:400 diluted samples: here, 50 μL of the 1:20 diluted sample was mixed with 950 μL of methanol/water (10/90, v/v).

When field samples contained very low concentrations (below LOQ) of analytes (specifically, amino acids), these samples were concentrated ten-fold. Five hundred μL of the original aqueous extract were transferred to an autosampler vial (Waters Corp) using an air displacement pipette. The samples were left in a chemical fume hood at 25 °C for one week to evaporate all the water. Once the water had evaporated, the analytes were re-suspended in 50 μL of 18 MΩ organic-free water. These autosampler vials were capped and sonicated in a 25 °C water bath for 15 min in degas mode. The 50 μL volume was transferred into a 100 μL PolySpring insert prior to LC/MS analysis.

## Results

*Sugars:* Using these developed methods, approximately 250 samples of aphid honeydews were analyzed and quantified. The ecological implications of these concentrations are reported in a partner study [15]. Xylose was not detected in any sample analyzed. For other sugars analyzed in aphid honeydew, concentrations ranged from not detected (ND) to 280.8 mg/L (fructose), ND to 163.2 mg/L (glucose), ND to 415.8 mg/L (sucrose), ND to 11.5 mg/L (trehalose), ND to 810.4 mg/L (melezitose), and ND to 13.6 mg/L (raffinose).

*Amino acids:* Approximately 200 samples of aphid honeydew were analyzed and quantified. Glutamic acid concentrations ranged from ND to 9.4 mg/L, ND to 53.8 mg/L (glutamine), ND to 7.9 mg/L (aspartic acid), ND to 14.1 mg/L (serine), and ND to 89.4 mg/L (asparagine).

## Conclusions

These developed methods successfully separated seven sugar analytes in under 7 min and five amino acids in under 10 min. Limits of quantitation ranged from 0.05 mg/L (melezitose) to 1.0 mg/L (fructose) for sugars and from 0.10 mg/L (glutamic acid, MRM mode) to 3.66 mg/L (asparagine, MRM mode) for amino acids. Sample preparation was fast and simple, requiring only the washing of foils used to collect aphid honeydew with hot (80 °C) water and sonication of samples prior to LC/MS analysis for both classes of analytes. No analyte derivatization was required and excellent chromatographic characteristics with well-resolved peaks for analytes with the same *m/z* ratio were generally observed. For samples that had high concentrations of sugars and amino acids, dilution (1:20 or 1:400) in water was required. To improve sensitivity in the analysis of amino acids, aqueous aphid honeydew extracts were concentrated 10-fold. For those studying honeydew-mediated interactions in the field, this technique allows for characterization of ecologically important amino acids and sugars.

## Declaration of Competing Interest

The authors declare that they have no known competing financial interests or personal relationships that could have appeared to influence the work reported in this paper.
